# Court as a health intervention to advance Canada’s achievement of the sustainable development goals : a multi-pronged analysis of Vancouver’s Downtown Community Court

**DOI:** 10.1186/s12992-019-0511-9

**Published:** 2019-12-18

**Authors:** Regiane A. Garcia, Kristi Heather Kenyon, Claire E. Brolan, Juliana Coughlin, Daniel D. Guedes

**Affiliations:** 1 University of British Columbia, Vancouver, Canada; 2 University of Winnipeg, Winnipeg, Canada; 3 Centre for Policy Futures, University of Queensland, Brisbane, Australia; 4 University of Manitoba, Winnipeg, Canada; 5 University of British Columbia, Vancouver, Canada

**Keywords:** Sustainable development goals, SDGs, Health and human rights, Harm reduction, Opioid crisis, Problematic substance use, Criminal justice system, Problem-solving courts, Community courts, Canada

## Abstract

**Background:**

The increase in problematic substance use is a major problem in Canada and elsewhere, placing a heavy burden on health and justice system resources given a spike in drug-related offences. Thus, achievement of Sustainable Development Goal (SDG) Target 3.5 to ‘Strengthen the prevention and treatment of substance abuse’ is important for Canada’s overall realization of the SDGs, including SDG 3 (Good Health and Wellbeing). Since 2008, Vancouver’s Downtown Community Court (DCC) has pioneered an innovative partnership among the justice, health and social service systems to address individuals’ needs and circumstances leading to criminal behaviour. While researchers have examined the DCC’s impact on reducing recidivism, with Canada’s SDG health commitments in mind, we set out to examine the ways health and the social determinants of health (SDH) are engaged and framed externally with regard to DCC functioning, as well as internally by DCC actors. We employed a multi-pronged approach analyzing (1) publicly available DCC documents, (2) print media coverage, and (3) health-related discourse and references in DCC hearings.

**Results:**

The documentary analysis showed that health and the SDH are framed by the DCC as instrumental for reducing drug-related offences and improving public safety. The observation data indicate that judges use health and SDH in providing context, understanding triggers for offences and offering rationale for sentencing and management plans that connect individuals to healthcare, social and cultural services.

**Conclusions:**

Our study contributes new insights on the effectiveness of the DCC as a means to integrate justice, health and social services for improved health and community safety. The development of such community court interventions, and their impact on health and the SDH, should be reported on by Canada and other countries as a key contribution to SDG 3 achievement, as well as the fulfillment of other targets under the SDG framework that contain the SDH. Consideration should be given by Canada as to how to capture and integrate the important data generated by the DCC and other problem-solving courts into SDG reporting metrics. Certainly, the DCC advances the SDGs’ underlying Leave No One Behind principle in a high-income country context.

## Introduction

This paper examines how the Downtown Community Court (DCC) in Vancouver addresses the health needs and realizes the full human potential of individuals, as proscribed under the Sustainable Development Goals (SDGs) agenda, of a local and marginalized segment of the Canadian population [1]. As in other countries [2], the increase in problematic substance use has become a major problem in many Canadian provinces, including British Columbia where the DCC is located [3]. Understanding the DCC’s framework and holistic practice may help other jurisdictions in Canada and elsewhere with similar struggles to design integrated strategies to more effectively address the underlying determinants of health and inter-related factors influencing criminal behavior. Indeed, we submit that the development of such community court interventions, and their substantive impact, should be reported on by Canada and other countries as an important contribution to national achievement of Sustainable Development Goal (SDG) 3 (Good Health and Well-Being), particularly SDG targets 3.4 and 3.5, as well as other cross-cutting targets under the broader SDG framework that coalesce with the social determinants of health (SDH) and systems strengthening (Table 1). In terms of the DCC, these could include targets found in SDG 1 (No Poverty), SDG 5 (Gender Equality), SDG 10 (Reduce Inequalities), SDG 16 (Peace Justice and Strong Institutions) and SDG 17 (Partnerships for the Goals).

**Table 1:** The operation of Vancouver’s Downtown Community Court can directly contribute to the achievement of targets in Sustainable Development Goal 3 (Good Health and Well-Being) and support achievement of inter-related goals


SDG Target 1.3: Implement nationally appropriate social protection systems and measures for all, including floors, and by 2030 achieve substantial coverage of the poor and the vulnerable.	
SDG Target 1.4: By 2030 ensure that all men and women, particularly the poor and the vulnerable, have equal rights to economic resources, as well as access to basic services, ownership, and control over land and other forms of property, inheritance, natural resources, appropriate new technology and financial services including microfinance.	
SDG Target 3.4: By 2030 reduce by one-third pre-mature mortality from non-communicable diseases (NCDs) through prevention and treatment and promote mental health and wellbeing.	
SDG Target 3.5: Strengthen the prevention and treatment of substance abuse, including narcotic drug abuse and harmful use of alcohol.	
SDG Target 5.1: End all forms of discrimination against women and girls everywhere.	
SDG Target 10.2: By 2030 empower and promote the social, economic and political inclusion of all irrespective of age, sex, disability, race, ethnicity, origin, religion or economic or other status.	
SDG Target 10.3: Ensure equal opportunity and reduce inequalities of outcome, including by eliminating discriminatory laws, policies and practices and promoting appropriate legislation, policies and action in this regard.	
SDG Target 16.b: Promote and enforce non-discriminatory laws and policies for sustainable development.	
SDG Target 17.17: Encourage and promote effective public, public-private, and civil society partnerships, building on the experience and resourcing strategies of partnerships.	

Using the DCC as a case study, the objective of our analysis is to examine the ways in which health and the SDH are addressed by a purpose-built court that implicitly aims to tackle the underlying SDH that lead to or are consequences of criminal offences fueled by addiction, poverty, mental illness, poor health, insecure housing, discrimination and rights abuses, as well as fractured social and familial networks. We therefore designed a study that consists of three inter-related research components: 1) documentary analysis of all publicly-available DCC reports and newsletters; 2) observational analysis of the court and how health and the SDH are addressed in practice; and a 3) print media analysis with a focus on how media addresses the DCC’s impact on client health and the SDH. Together, these methods allow us to gain deeper insight into how the DCC’s design and daily operation intersect with and could inform Canada’s SDG agenda and, specifically, SDG 3 achievement.

The Background section sets out our understanding of the context in which the DCC has emerged and operates in Canada, and within the context of problem-solving courts more broadly. Problem-solving courts form part of the cluster of “specialized courts”, which can include community courts, such as the DCC, mental health courts, domestic violence courts and drug courts, as discussed later in this paper.^1^ We situate the DCC’s emergence in the literature on problem-solving courts, including crosscutting public health literature [4]. We argue that the DCC is not only a legal intervention, but also an important health and human rights intervention. The limited existing research on the DCC employs recidivism rates as the key metric [3], therefore, overlooking the DCC’s important contribution as a health and human rights intervention. This is unsurprising; much of the literature around problem-solving courts investigates the impact of these courts on recidivism rates [5–12]. Research on recidivism is important for both substantive and practical reasons. The existence of such courts can be controversial, and thus the efficacy and cost-effectiveness of problem-solving courts often require an evidence-base to justify ongoing investment by government. Yet, an exclusive focus on effectiveness, particularly through recidivism rates,^2^ can shift the framing of the person at the heart of the judicial process – and the inherent human dignity of that individual – into the quantifiable or numerical. We fear this approach can detract from a health and human rights approach to harm reduction advanced by the UN Special Rapporteur on the Right to Health [4], wherein individual human dignity is central [13].

There is also risk that by employing a recidivism lens, the metrics and language of recidivism may skew research to appear to align with the recovery agenda. The recovery agenda, according to Paylor, “can be seen as reinforcing negative stigma around drug use” as it suggests that “only those willing to ‘get clean’ deserve the benefits offered by society” [14]. Alternatively, the harm reduction model is a growing social epistemological and clinical approach that also links to broader debates on the sociology of health and human rights-based approaches to drug control [4, 15, 16]. While the harm reduction, community court intervention, and health and human rights nexus is not the focus of this paper, we acknowledge the growing literature at that crossroad which overlaps and anchors the implicit harm reduction approach further adopted by our research team [17–20].

## Background

### History of the DCC

In September 2008, Canada’s first community court, the DCC, was launched in Vancouver’s Downtown Eastside.^3^ The Court was created through an innovative partnership between Canada’s Ministry of Justice, the Provincial Court of British Columbia and 14 health and social services agencies subsequently co-located in the courthouse [21]. In the early to mid-2000s, Vancouver was hit hard by overdose fatalities [22], with a particular growth in overdoses from opioids such as methadone, morphine, codeine and oxycodone. The effects of this problematic substance use increase were experienced most strongly in Vancouver’s Downtown Eastside where overdoses were concentrated, and where drug use was linked to communicable disease transmission and criminal activity, both placing heavy burdens on communities and government resources [23]. The health system responded by opening ‘Insite’ in 2003; North America’s first supervised injection site, which helped to reduce drug related mortality [24]. Insite took a harm reduction approach by providing clean needles, a safe medically supervised place to inject, first aid services and access to treatment options. Vancouver’s criminal justice system struggled to manage a co-occurring increase in drug-related crime [25].

In 2004, the British Columbia Justice Review Task Force established a Street Crime Working Group to scope and propose strategies to address street crime and disorderly behavior in downtown Vancouver. The Working Group held public hearings and consultations with diverse stakeholders, including all levels of the justice system, social service agencies, community organizations, and affected communities. It also undertook data analysis and a review of approaches from other jurisdictions, particularly the United States [26]. This resulted in the “Beyond the Revolving Door Report” [26]. This report included comprehensive recommendations that proposed greater integration and collaboration among actors in the criminal justice system, health system and social services, in order to respond to street crime and address the underlying complex needs of persons who repeatedly commit street and drug-related crime [26].

In 2006, the federal and provincial governments together with the Office of the Chief Judge endorsed the report and created a planning committee to design the DCC. The planning committee engaged with other jurisdictions with successful community courts, held public forums and discussions with affected communities, Indigenous organizations, justice system staff and service providers in the DCC catchment area [26]. This catchment area included several adjacent neighborhoods: the Downtown core, the Downtown Eastside, Stanley Park, Strathcona and the West End. The Downtown Eastside is one of Canada’s poorest neighborhoods, with a median total income of $20,617 (compared to Canada’s $34,204) [27].

In 2007, the committee received funding to implement a pilot project to develop the physical space for the courthouse, and the DCC opened in 2008 [21]. The DCC was created as a purpose-designed, community-based problem-solving court that uses an intersectoral, multi-stakeholder approach to address the needs and circumstances of the individuals (‘clients’) who appear before it. Where deemed appropriate, judges employ diverted sentencing including community service alongside a personalized plan that may include housing support, social service access, employment counselling and links to culturally appropriate services.

### History of problem-solving courts

Drug courts, one of the first types of specialized courts, originated in Miami in the United States in the late 1980s to respond to the increasing number of felony drug charges in the Miami-Dade County area [28–31]. The model has since expanded to become a critical part of the problem-solving court “movement” in many countries [31]. Such courts are engaged in “therapeutic jurisprudence” [32] in which legal rules and procedures have therapeutic effects “whether intended or not” [33]. Typically, problem-solving courts do not have trials with the judge typically playing a role of facilitator rather than arbitrator [34]. Instead of engaging in an adversarial process, judges provide court-supervised treatment to certain groups of offenders seeking in so doing to address the underlying causes of criminal behaviour [35–37].

Problem-solving courts are an important tool to break the revolving door pattern of drug-fueled offending and to reduce prison costs and inmate numbers. Such courts may impose mandatory drug treatment combined with drug testing to ensure participants continue to adhere to the court program; but this is not the case of the DCC, where treatment is arranged on a voluntary basis. Problem-solving court models use “legal leverage”, broadly defined as the use of legal authority to promote treatment adherence and good health and well-being [38] and to bring to life the principle of “Differentiated Case Management” in court administration [39]. The referral of an individual to a specialized court program may occur at different points in the justice process depending on jurisdiction. Often, such courts are harnessed post-arrest “as an alternative to traditional criminal justice processing”, avoiding the use of prisons as the first point of sanction [40].

Overall, the literature on problem-solving courts is multi-disciplinary, principally spanning the legal, medical and public health disciplines. As the literature on problem-solving courts has expanded in the past two decades, there has been a shift in focus from a ‘war on drugs’ approach to drug addiction and drug-related crime, to a comprehensive public health and well-being response to court participants and their families [41, 42]. Therefore, much of the research on these courts is grounded in public health, social science and legal methodological perspectives and approaches [34]. Missing from this literature is a right to health approach, or a consideration of such courts and the well-being participants through the lens of health and human rights. What is also missing is consideration of how these courts may support countries, especially high-income countries, to ‘activate’ their commitment toward achieving Agenda 2030 and its 17 SDGs.

### What do we know about the effectiveness of problem-solving courts?

Since 2000, the body of literature on the operation of drug courts in high-income nations has grown, although it continues to focus mainly on the United States. Much literature on these courts investigates their efficacy through a cost-effectiveness lens [9, 43, 44], while also highlighting the multi-dimensional net-benefits for court participants and their families [45], and examining family reunification and child welfare outcomes [46–49]. There is also a great deal of research that demonstrates the effectiveness of such courts in lowering recidivism, including among young people and women [5–12].

However, calls remain for more rigorous cost-effectiveness evidence, research on the impact on reduced recidivism over the longer term, and family and child welfare outcomes [50–52]. There is also concern that some countries created problem-solving courts too quickly without robust evidence on their effectiveness [52]. Further concerns centre on the ongoing need to develop court programs and interventions in consultation with ethnic and other minorities [16], as well as for these courts to be adapted in different jurisdictional settings to reflect local judicial, criminal justice, public health, social and cultural contexts [53–57].

The public health literature examining problem-solving courts’ impact on health and well-being focuses on four main areas (1) the role of healthcare professionals and how they can optimally support court participants [58–60]; (2) how the connection between courts and health and social services can be strengthened [61]; (3) how to improve treatment initiatives [62–64]; and (4) healthcare related experiences of court participants [65]. The public health literature also emphasizes the important role crosscutting Universal Health Coverage plays for low-income court participants, especially in the United States [66]. Several studies examine the unmet health and well-being needs of court participants including needs related to chronic medical condition diagnosis and management, and improved health care planning [67–69]. Additional literature explores the sexual and reproductive health needs of court participants, with studies examining interventions to decrease HIV and sexual risk taking behaviours [70, 71]. Studies on the mental health of court participants are also prevalent, although as with studies on sexual and reproductive health, further research is recommended. Mental health-related studies review psychological stressors that exacerbate addictive behaviour resulting in potential recidivism, mental health screening and interventions in court settings, the effectiveness of therapeutic communities such as group counselling and cognitive-behavioural therapies, and psychosocial benefits of these courts on participants [72–76].

The public health literature also shows that the complex health, well-being and recidivism issues faced by court participants are often inherently structural, and therefore interconnect with the SDH such as employment, education, training and housing [77, 78]. Researchers consequently call for a multi-pronged, multi-agency holistic response by such courts, as opposed to a biomedical drug intervention or siloed treatment approach [79]. When such courts are first set up, the ‘criminal justice’ purpose of the court and the need for a greater health and well-being approach toward intervention activities is cited as a source of tension between court actors and stakeholders [80]. However, participants have identified that caseworker support is a crucial element for successful graduation from problem-solving courts [81] alongside the attachment of “supportive services” to these courts such as debt and domestic violence counselling, financial management, child care and parenting information and mentoring [82]. That said, a 2015 study on what court participants saw as successful intervention factors found frequent contact with the court judge and random drug testing to be crucial [83], while another study found women participants credited their recovery and successful completion of the court designed plan program primarily due to a mix of fear of punishment and program structure [84–86].

Returning to the Canadian context, the literature examining problem-solving courts is largely from other provinces and not British Columbia [16, 54]. There is also limited literature on the DCC. The only published study on the DCC is a quasi-experimental study that examined the impact of the DCC in reducing recidivism in the Court’s geographic catchment area [3]. That study, by Somers et al., compared recidivism rates among chronic offenders assigned to the DCC’s Case Management Team (an interdisciplinary team to assist with individuals’ rehabilitation) with the recidivism rates of chronic offenders in the provincial criminal court that received traditional justice responses [3]. The DCC’s Case Management Team includes inter-disciplinary expertise and connects offenders to community resources and monitors their progress. Similarly to studies in other jurisdictions, Somers et al. found that DCC clients connected to the Case Management Team had significantly greater reductions in overall offending in comparison to those who were not connected with management teams [3].

## Methods

In the tradition of ‘law and society’ scholarship [87], this study focuses on the interrelationship between law and social context employing an interdisciplinary methodological toolkit. With particular attention to health, well-being and the SDH, we designed and conducted a multi-pronged case study on the DCC by: 1) analyzing DCC evaluation reports and newsletters, 2) observing DCC hearings [88], and 3) examining press media representation of the DCC. Our research is based solely on publicly available data. We included publicly available reports and media in our documentary and media analysis, and observed public hearings of the DCC from the public galleries with the knowledge of the DCC (February 11, 2019).

### Document analysis

The document analysis aimed to determine how the DCC framework engaged with health and the SDH. Two complete sets of publicly available records were located and analyzed: evaluation reports and DCC newsletters. Using content analysis [89], we began by examining how the DCC reports address health and the SDH and how they position the Court’s role in relation to those factors. We then examined the DCC newsletter content employing a similar content analysis approach, with a focus on how the DCC engages with health and the SDH in presenting its objectives and achievements in these publications.

### Court observation

Our Court observation aimed to understand how, when and why the Court raises and addresses health and the SDH in practice [88]. Court observation is a form of systematic observation and was employed by the research team to discern how ideas or concepts around health and the SDH are addressed, engaged and incorporated into sentencing [90]. Although the Court record is available online, observations were made in situ to gain a better understanding of context. We conducted non-intrusive naturalistic observation [91] with two legally-trained members of the research team (RG, DG) attending Court on alternate days and noting types of crime, sentencing outcomes, and self-declared demographic information. Seated in the public gallery, the team members observed 78 h of hearings over 13 days between February 14 and March 8, 2019.

Even though the DCC hearings are open to the public and decisions are public record, we undertook a number of precautionary measures to gain entry to the DCC and maintain client confidentiality. For example, we closely corresponded with DCC staff and judges to engage in court observation and answered their questions with respect to research aims, data collection, and data dissemination plan. Research team members made no contact with clients. Although names are available through the online record, we chose neither to identify judges, prosecutors, lawyers or clients nor to list specific hearing dates in order to offer a degree of confidentiality to Court actors and vulnerable populations.

In terms of data collection, detailed notes were taken of all mentions of health and SDH with direct quotations where possible. Notes were entered into two structured grids, titled ‘sentencing hearings’ and ‘adjournment hearings’, and hearings were numbered in chronological order according to Court appearance to facilitate comparative analysis and quotations. In total, team members RG and DG observed 86 sentencing hearings and 375 adjournment hearings. Because adjournment hearings were brief and lacked information in our area of focus, we decided to limit our analysis to sentencing hearings only, with KHK thematically coding the observation data from the sentencing hearing grids [92]. There was iterative discussion throughout the process and agreement among team members as to emergent themes and findings.

## Media analysis

Finally, the research team (led by JC) conducted a media review to examine how the Court is depicted in Canada’s mainstream print media. We identified key local, provincial and national publications using the search term “Downtown Community Court” in each publication, as well as the Google search shortcut (term site: (media outlet i.e. Globeandmail.com)) for January 1, 2008 to February 1, 2019. Our media survey slightly pre-dates the Court’s launch, as we wanted to capture media coverage in the lead up. We started by a general Google search, followed by a university library portal search to locate any other media sources reporting on the DCC. A total of 60 articles were located using these methods, of which 21 contained a detailed rather than passing reference to the DCC and were included in the content analysis.

## Results

### Evaluation reports

We analyzed the two publicly available and internally prepared documents providing an “official” version of the structural framework of the DCC: the Interim Evaluation Report produced by the Ministry of Attorney General and the Ministry of Public Safety and Solicitor General, published in 2010 (“Interim Report”) [93]; and the Final Evaluation Report produced by the DCC’s Executive Board, published in 2013 (“Final Report”) [21]. Explicit reference to health was recurrent throughout both documents, with 98 references in the Interim Report and 35 references in the Final Report. Both reports, however, made clear that “at its core, the DCC is about testing new ways to reduce crime and improve public safety” [93], with plentiful reference to the DCC been driven by the goals of improving efficiency (i.e., reduce court appearance and processing times) and ensuring the safety of the public [21, 93].

We found that in the Interim and Final Reports health intersected with the DCC’s goals in two contexts. First, it intersected in relation to the health of the DCC’s target population: “chronic offenders whose offences are associated with problematic substance use, mental illness and poverty” [94]. Second, we saw connections with respect to clients’ individual management plan; that is, health and SDH strategies to address the risk to reoffend and address their underlying needs leading to criminal behaviour [21].

We further found that the Interim and Final Reports on the DCC often referenced the SDH. Housing was the most referenced SDH with 31 references in the Interim Report, and seven references in the Final Report. Housing, as well as other SDH, were discussed in relation to the DCC’s goals in two ways: to provide context for the criminal behavior (e.g., lack of housing, employment, social assistance), and to indicate the types of services integrated into the DCC’s multidisciplinary Case Management Team.

### Newsletters

We located and analyzed six DCC newsletters. The purpose of the newsletters was to inform the public about program updates, community services, client profiles and achievements and provide judge’s perspectives. The newsletters contained occasional references to how the DCC’s programs intersect with health and the SDH. For example, in the judge’s perspective section of the Fall 2012 newsletter, Judge Gove is quoted on how the DCC’s Mental Health Program “has allowed offenders with mental health disorders to connect with housing and community mental health services resulting in many cases no longer proceeding by way of criminal charges”. Another example is reported in the Spring/Summer 2012 edition, with an article reporting on a DCC program that supports food security tilted “Community Based Experiential Learning”, a program in which the DCC hosts fourth year students from the University of British Columbia’s Faculty of Land and Food Systems. In this particular newsletter, it was reported that the students developed a cookbook for the “Healthy Eating” program, which is “a low-barrier program teaching clients how to cook healthy, nutritious meals in a SRO [Single Room Occupancy rentals in hotels in the Downtown Eastside], on a single burner for under $5”.

## Court observation

Our observed sample consists of substantial judicial decisions, primarily a sentence or a judicial interim release, in the cases of 86 different defendants – referred to as “clients” in the DCC system.

### Client demographics and common criminal offences

We found that males were over-represented in the sample, accounting for 87.2% (*n* = 75) of all clients. Twenty-six clients were held in custody, attending Court either in person or through videoconference; twelve of these were overnight arrests. Three clients did not speak English and were assisted by interpreters.

In our observational sample, the most common criminal offences brought before the Court were theft and breach of court orders, and virtually all clients had a previous breach. Fifty per cent of clients (*n* = 43) appeared or had previously appeared before the court for charges of theft under $5000. Thirty-seven per cent of clients (*n* = 32) were in Court due to breaches of bail, probation order, recognizance or undertaking. Offences involving violence or uttering threats came next, with 17.4% of clients (*n* = 15) charged with assault (including with weapons or causing bodily harm), while 12.8% of clients (*n* = 11) were charged for uttering threats. Just over 9% of clients (*n* = 8) were charged with matters involving domestic violence. There were 18.6% of clients (*n* = 16) charged with mischief; 5.8% of clients (*n* = 5) charged with possession of stolen property; four charged with unlawful possession of identification documents; four with unlawful possession of weapon; three with break and enter; two with driving while impaired; and one with possession of controlled substance. We found it is not uncommon for clients to appear before the Court with multiple charges, particularly in combination with a breach of Court order.

### Health and social determinants of health in sentencing hearings

References to the client’s health or to the SDH were made in all but one of the 86 hearings resulting in judgments. Counsel and judges used such information to provide context and understand triggers for offences. Judges also referenced these factors in designing management plans (for deterrence, treatment and community restitution) and in justifying sentencing. In 67 of the 86 hearings observed, judges explicitly recognized health (e.g., mental illness, addictions, other health conditions) and the SDH (e.g., homelessness, poverty, intergenerational trauma and social isolation) as the underlying causes of criminal offences and aimed to address these issues through therapeutic sentencing and engagement with health and social service agencies.

Challenges relating to client housing were referenced in 32 hearings, with 19 clients indicated by the Court as homeless and an additional three referred to as having previously been homeless. Other mentions of housing included impending eviction and precarious arrangements such as living in hotels, squats or with friends and family. Homelessness and housing were referenced to provide context, indicate the ability to access services and to explain motivations. In one hearing, lack of housing was noted as a barrier to needed care, with counsel stating that “counselling is difficult” as the client was moving between shelters [client 5]. In several cases, the lack of secure housing was linked to a ‘disorganized life’ with several references across our data to clients consequently struggling with adherence to medication regularly or relapsing into addiction. For example, a defence lawyer explained, “[my client] is at a shelter at Cordova Street. His lack of participation [in the DCC’s Mental Health Program] is due to his disorganized life.” [client 62]. In another case, a judge noted, “I am familiar with [the client’s] long history of mental health and of not taking his medication while on the streets in addition to street drug use.” [client 4].

In many instances, judges also recognized homelessness as a trigger for Court order breaches; breaches represented the vast majority of observed hearings. Homelessness was linked to failure to appear in Court or maintain contact with probation officers leading to a reinforcing cycle of homelessness and criminal behaviour. In one such hearing, Crown counsel explained:

“[The client] has 116 convictions and 75 breaches. [The client] goes back to these [no-go] areas because this is where he lived before. His situation is a revolving door. A referral [to the DCC’s Case Management Team] would offer a more supervised unit, assistance with homelessness, basic income, and counselling condition”. [client 9].

The Crown counsel, in this case, indicated that police had suggested “trying something new” [client 9] with more structure and support with housing and access to healthcare. The client indicated he was willing to accept help, noting: “I am homeless. I want to be far from drugs, far from trouble. I am willing to work with everybody here. I am ready”. [client 9]. In sentencing this case, the judge acknowledged the “revolving door pattern”, noting: “you showed a lack of respect for Court orders. But I see a pattern of homeless and addiction here.” [client 9]. In this illustrative case, to assist with the client’s multiple needs, the judge ordered the client’s judicial interim release from overnight custody [clients waiting for a bail hearing] combined with one-year probation and Case Management Team supervision.

Poverty and access to income surfaced frequently as an additional SDH affecting the Court’s clients. There were nine references to unemployment or loss of employment, with six clients described as being on social assistance of some form, and four references to clients in need of access to social assistance. In several instances, judges explicitly linked poverty and offences (often in relation to theft under $1000). One judge explained: “your offence was motivated by poverty rather than addiction”, deciding: “I will not have you reporting, because you have a job and I don’t want you to lose your job”. [client 78].

In 72 of the 86 hearings, the presiding judge referred to problematic substance use as critical contextual factors. The range of drugs to which clients were addicted included pain medication, crack, heroin, cocaine, fentanyl, crystal meth and alcohol. In some cases, clients reported losing employment, relationships and connections to their children through addiction, while in others such losses precipitated addiction. For example, in one hearing the client’s lawyer explained: “[He] lost his job in the oil industry. He is now homeless and has serious addiction issues.” [client 79]. In other cases, physical injuries impeded physical forms of employment, and resulted in a prescription for painkillers that led to opioid addictions.

Mental health issues beyond addiction were also frequently referenced in the DCC. Mental health issues recorded in our data included depression, post-traumatic stress disorder, anxiety, bipolar disorder, drug-induced psychosis, attention deficit and hyperactivity disorder, schizophrenia, self-harm, and general references to mental illness, or to being detained under British Columbia’s Mental Health Act. Mental illness issues were contextualized as resulting from life disruptions and traumas, including childhood abuse, sexual assault, inter-generational trauma of Indian Residential Schools, relationship breakdowns and the death of close family and friends. Mental health issues were also referenced as contributing causes to family estrangement, job loss or difficulty maintaining employment, and conflict with the law.

In many hearings featuring addiction and mental health, the judge, in consultation with defence and Crown counsels, developed a plan to access treatment as part of a suspended sentence. Counselling was often recommended, as was engagement with affiliated services, including the DCC’s Case Management Team and Mental Health Program, residential treatment programs, detox programs and hospital-based programs. The DCC’s Case Management Team alone was referenced 72 times in the cases observed. As the diversity of programs suggests, recommended treatment did not follow a prescribed modality, but was context-specific and included recovery-oriented detoxification programs and harm-reduction programs of drug substitution involving drugs such as methadone and Suboxone [14, 16].

In sentencing, DCC judges often considered the reason for the crime, the health of the client, their comfort with existing service providers and their mental and physical capacity to perform community service. For example, in one case the judge noted that the client had been working with the DCC’s Case Management Team, and did not add counselling in the sentence, as the client was comfortable continuing with the DCC’s team. In considering a client’s ability to adhere to drug treatment, a judge ordered the client change the location of his methadone prescription by imposing an area restriction condition; to stay away from the Downtown Eastside neighborhood, an area with “too many temptations” for this particular client. In another example, the judge acknowledged a client’s progress on the methadone program while in custody, but doubted his ability to abide by probation conditions, and thus decided to keep the client in custody to better manage his addiction. In considering community service, a judge noted physical capacity stating, “I saw you hurt your back. I will not sentence community work”. [client 57].

In 27 of the 86 cases observed (31%), clients expressed a desire for drug treatment, while in eight cases (9%) clients expressed an unwillingness to receive treatment. One client living with bipolar disorder and addiction was described as “determined to get help” and willing to engage with the DCC’s Case Management Team, explaining her offence (theft under Can$5,000) stating: “I did things in desperation. I need help. I need to get well.” [client 13]. In some instances clients were “not keen on counselling” [client 34] and on two occasions, clients were not interested in drug treatment at all, which generally resulted in mandatory counselling and treatment provisions not being included in sentencing.

Other health issues that arose in sentencing included chronic illness (e.g., diabetes and HIV), physical injury, mental injury and cognitive impairment. On two occasions, the presiding judge did not include mandatory counselling in sentencing where clients faced challenges in benefitting from treatment due to health conditions such as brain injury and depression.

Social and familial disruption and the role these connections played in healing were prominent themes throughout the hearings. Many of those before the Court experienced disruption of family and social networks and intergenerational trauma. For example, Indian Residential Schools were referenced as a contributing factor in four hearings - in one case the client had attended residential school, while three clients had parents who attended such schools. In presenting the client’s background in each instance, the defence linked these schools, which removed Indigenous children from their families and communities, punished cultural expression and the speaking of Indigenous languages, to intergenerational trauma. In one of these cases, the client was described as having “no family connections”, “restricted access” to his children and was now “seeking assistance to deal with childhood trauma.” [client 57]. In several other cases, social and familial connections and disruptions were reported in relation to international or domestic migration. Several clients had come to Vancouver from other countries including the Philippines, El Salvador, Egypt and Fiji, either alone or with limited family. One defence counsel noted in reference to his client’s isolation: “He is from Fiji. He is alone, no family and is isolated.” [client 78]. Isolation and distance from family were also reported with respect to migration from within the province and country, with DCC clients from Ontario, Manitoba, Alberta, Nova Scotia and other parts of British Columbia.

While family breakdowns and estrangement were often mentioned, the Court also referenced familial connections as sources of support, strength and motivation. In one case where addiction followed divorce, it was noted that the client’s mother and daughter were seated in the gallery, with defence counsel stating, “[the client] is motivated to undergo counselling because he wants to become clean from addiction and be the good father he once was.” [client 41]. In another case, the defence counsel similarly stated: “[the client] has a 9-year-old son and is motivated to be good for his son’s birthday next week.”[client 63].

Judges often showed a deep understanding and respect for clients’ life circumstances in the hearings. For example, comments included “I acknowledge you struggle with many barriers” [client 15], “I recognize that you had a hard time” [client 1], and “I am very motivated by your recovery” [client 16]. One judge stated to a client:

[Y] ou have a period [where] you were doing well. It is encouraging the CMT is willing to take you back. 30-day conditional sentencing is nicer, and then you are not in custody. I hope you are focused. I want you to focus on your recovery.” [client 34].

This understanding however, was not without limits. Acknowledging that poor behaviour was “obviously [ …] due to addiction”, while determining probation, one judge noted: “if you breach [conditions] again, you won’t get [probation] from me again, but instead will receive a custodial sentence.” [client 6]. In another case, while considerate to the client’s adverse circumstances, a judge noted that while the current sentence was probation, “from here [the next step is] you go to jail” advising the client, “you need to engage with the [DCC’s] Case Management Team. They have not given up on you. Participate in programs: detox, counselling.” [client 59].

In cases of domestic violence, the measures DCC judges employed to protect the victim included orders of no-contact or permissive contact where the client was mandated to leave the victim immediately upon request. In such cases, discussions among the judge, counsels, clients, etc., typically revolved around the management plan to keep the victim safe and involved reference to the DCC’s Victim Services. In these cases, the violent behavior of clients was considered in terms of the victim’s safety, with the judge deciding in one such case, “I will allow permissive contact incrementally, [and] once you take steps toward the right direction [couples’ counselling], I will determine reporting for the purpose of counselling.” [client 59].

### Media survey

The findings from our content analysis of the mainstream print media on the DCC reflected many of the themes and issues that emerged from our Court observation. Of the 21 media articles examined, 20 articles mentioned health in some capacity, primarily referencing addiction and mental health. The SDH were addressed in 18 articles with a particular emphasis on housing and social connectivity.

Articles included interviews with judges and profiles of clients. A 2016 Canadian Broadcasting Corporation report featured a lengthy explanation from a DCC judge on the Court’s intake procedure, noting:

“[Judge] [Name] explained that when defendants first come to the court, they're given a needs assessment by one of 14 on-site service groups, which range from mental and physical health practitioners to probation officers who can help with housing and income assistance.” [94]

This example provides a number of insights into the DCC’s operation and approach, including services offered at the courthouse, information sharing to provide context concerning the underlining factors leading to crimes, mentions of received medications, addiction-related concerns, the need for additional services to meet health needs, and the need to understand client’s overall health.

SDH as factors influencing drug-related crime also arose in pieces profiling clients. For example, a Globe and Mail newspaper article of 2019 examined how “poverty, family breakdown and problematic substance use, can impact one’s life” [95]. This article also referred to how social support together with the DCC’s approach to drug-related crime could turn clients’ lives around. The client featured in the article explained that during one of his appearances, a judge told him that he had potential, and “[t] hat hit me hard. It planted a seed in me.” [95]. Other articles referred to SDH by offering insights into how these factors related to the mission and aspirations of the DCC, quoting Judge Gove, one of the founders of the DCC as saying: “I’m an idealist. But I’m not naïve”, acknowledging that political will and cooperation among all stakeholders are key to the success of the DCC’s ambitious health-related mission [96].

Consistent with our findings of the DCC Evaluation Reports set out in the first part of the Results section, we found that efficiency (i.e., reduced court appearances and processing times) was another prominent theme of media coverage. Indeed, the DCC Reports prompted media coverage, with a majority of articles include in our analysis published after the release of the DCC’s Final Evaluation Report in 2013. One such article noted: “the results of the efficiency evaluation indicate that the DCC had a neutral impact on efficiencies.” [97]. The same piece refers to the DCC’s efficiency and health and SDH integrated approach, citing Chief Judge Thomas Crabtree’s remark that when underlying needs leading to drug-related crimes are met through the DCC, “reduction in recidivism is significantly greater than that at a traditional court.” [96]. Another piece refers to the DCC’s efficiency and health & SDH integrated approach, citing a Chief Judge’s remark that when underlying needs leading to drug-related crimes are met through the DCC, “reduction in recidivism is significantly greater than that at a traditional court”. [97]. One piece, however, raises questions as to whether the DCC’s integrated services are “well-meaning Band-Aids”, with the author asking whether referrals to “more flexible socio-health authorities” would be more appropriate [98].

## Discussion

This study investigates how health and well-being were addressed and framed in the DCC’s framework and daily operations by Court judges, counsels and clients. We employed a multi-pronged investigative approach examining: (1) publicly available DCC documents, (2) coverage by the print media, and a series of (3) Court observations examining health-related discourse and reference.

In the documentary analysis we found explicit and recurrent references to the health of DCC clients in the reports in comparison with the newsletters. One hundred and thirty-three (*n* = 133) direct references to health were made in the two reports analyzed. The SDH were also discussed, with housing the most frequently raised. The newsletters’ reference to health was more infrequent and nuanced and mainly referenced SDH.

In answering how the Court’s foundational documents along with Court and media discourse frame health and the SDH, the analysis showed that health and the SDH are framed as instrumental for achieving the DCC’s goals of efficiency and public safety. Health and the SDH are hence associated with both the target population and the Court’s integrated interventions to address the underlying factors influencing their criminal behavior. Our analysis indicates that the structural framework that establishes and under which the Court operates requires continuous considerations and effective access to health and SDH-related services for accomplishing its foundational goals.

The Court observation component of this study showed how DCC judges take health and the SDH into consideration in sentencing. Of the 86 hearings observed, reference to the client’s health, or impact or intersection with SDH factors vis-à-vis the client’s individual circumstance in the justice system, was raised, particularly to provide context and understand potential triggers for the conduct of illegal activity. In terms of the SDH, housing issues featured prominently in hearings just as within the DCC Reports. Clients’ poverty, income and employment, social and family connectivity also featured prominently. Court observation also illustrated the diverse health issues experienced by DCC clients with judges often directing clients, as part of their sentencing, to engage with affiliated health and social services. As previous studies elsewhere suggest [4, 15, 45–52], our research indicates that in assessing the outcomes of the DCC, further studies need to pay close attention to the essential role played by effective supportive teams, treatment initiatives, and experiences of Court actors and participants.

With respect to the media analysis, of the 21 media articles examined, 20 articles directly mentioned health, primarily referencing addiction and mental illness. The SDH were addressed in 18 media articles with a particular emphasis on the importance and challenges of housing and social connectivity for DCC clients. We found that the print media particularly focused on the efficiency of the Court, in particular recidivism, showing unfamiliarity with the critical role played by health and intersectional actions on the SDH to advance the Court’s ultimate success of reducing recidivism and court processing times.

It is important that we acknowledge that this study has a number of limitations. In terms of the court observation component of this research, this is a discrete, small-scale study that relies on 13 days of court observation of sentencing hearings. Further research, ideally longitudinal, is needed to examine whether and how judges, and the DCC as a whole, continue to carry out the task of balancing client’s health-related needs and the public’s safety over time. Indeed, further research that is grounded in a rights-based approach could investigate the extent to which the Court recommendations affect people’s lives, and improve their health and well-being. Further studies could also support the determination of appropriate metrics to evaluate the DCC and determine appropriate ways to communicate these findings to the broader community. Such research could be conducted through a rights-based lens in which the fundamental human dignity of the individuals at the heart of the DCC justice process is recognized and promoted.

Our findings suggest that the DCC is an important health intervention to advance the SDG agenda. Our data indicate the DCC regularly acts to improve clients’ health and well-being by connecting them to healthcare, social and cultural services. As a result, the DCC offers a promising context-sensitive balance between the health needs of vulnerable and marginalized populations and the public need for community safety.

## Conclusions

The DCC’s health-related impacts and activities, if the data are appropriately captured through both quantitative and qualitative means, could provide an important source of evidence for the Canadian Government to report on its SDG 3 achievement. This data could be particularly useful with reference to the Canadian Government’s commitment under SDG Target 3.5 to ‘strengthen the prevention and treatment of substance abuse, including narcotic drug abuse and harmful use of alcohol’. Yet such data could also significantly contribute to the measurement and monitoring of Canada’s achievement of at least five other SDGs that relate to the determinants of health and systems strengthening, namely SDG 1, 5, 10, 16 and 17. Certainly, the SDG agenda seeks to redress the multi-dimensional impacts of poverty, poor health, and inequities and inequalities in all countries (SDGs 1, 5 and 10), low and high-income alike, with the over-arching goal that ‘No One Will Be Left Behind’ [1]. Canada committed, when signing on to the SDGs in September 2015, to “endeavor to reach” population segments within its borders who are “the furthest behind first” [1]. DCC clients, and the clients of Canada’s other problem-solving courts, should thus be a strong focus in the Canadian Government’s contextualization and operationalization of the SDG agenda at home.

The growing problematic substance use has been detrimentally affecting individuals, families, communities and government resources in Vancouver and other jurisdictions within and beyond Canada’s borders. To achieve the 2030 Agenda for Sustainable Development and its 17 SDGs, and particularly SDG 3, countries must address both the impacts of and the underlying conditions leading to problematic substance use, and drug-related crimes; consistent with a harm reduction and health and human rights approach. Our study contributes new insights on the effectiveness of the DCC as a means to integrate justice, health and social services for improved health and community safety. In so doing, it highlights the DCC’s rich potential to serve as an explicit SDG lever for impactful local change.

As challenges to health and social equality advance in Canada and elsewhere, questions of how resources should be allocated are of utmost importance as they lay the necessary groundwork to give effect to the SDGs’ principle of Leave No One Behind. A new focus on service integration and coordination among the justice, health and social systems and sectors is needed to advance the rights of marginalized populations in Canada. Canada’s commitments under the SDG agenda and the DCC intervention should serve as both the inspiration and vehicle to bring about this much needed multi-sectoral, rights-based policy and planning shift.

## Data Availability

The paper contains de-identified data to ensure anonymity of all actors, excepting cases where an individual is identified in public records such as media reports. By making the raw data publicly available, it could potentially allow cross-reference with court public record allowing identification of the hearings and actors observed. The dataset can be made available upon request.

## Footnotes

1 While the *terms* “s*pecialized court”, “problem-solving court” and “community court”* are conceptually distinct, we use the terms *interchangeably* when referring to the DCC in this paper.

2 Other court effectiveness factors include reduced court appearances and case processing times.

3 For the purposes of this article, we refer to the DCC as a community court because Vancouver also has a separate, specialized drug court. Vancouver’s drug court can impose mandatory drug testing and treatment, which is not the case at the DCC, where client action and uptake of treatment services is voluntary.
